# Patient-centredness in primary care walk-in clinics for refugees in Hamburg

**DOI:** 10.1186/s12875-023-02060-2

**Published:** 2023-05-06

**Authors:** Josephine Nana Hill, Katarina Krüger, Sigrid Boczor, Thomas Kloppe, Olaf von dem Knesebeck, Martin Scherer

**Affiliations:** 1 Institute of Medical Sociology, University Medical Center Hamburg-Eppendorf, Hamburg, Germany; 2 Department of General Practice and Primary Care, University Medical Center Hamburg-Eppendorf, Hamburg, Germany

**Keywords:** Refugee, Video interpreter, Patient-centred, Shared decision-making, Empathy, Patient enablement, General practice consultation, Primary care walk-in clinic

## Abstract

**Background:**

The huge increase of refugees to Germany caused a great challenge to the health system. We aimed to examine the level of patient-centredness in medical consultations with refugee patients, aided by video interpreters in primary care walk-in clinics (PCWC) in Hamburg.

**Methods:**

Videotaped consultations (N = 92) of 83 patients from 2017 to 2018 were analysed. Two raters used the Measure of Patient-Centered Communication (MPCC) and the International Classification of primary care (ICPC-2). MPCC scores with regard to patients’ reason for seeking medical care and the procedures taken were explored using variance analyses adjusted for age, gender, and the duration of the consultation. The duration was further explored by Pearson correlations.

**Results:**

Patient-centredness of all consultations on average was 64% (95% CI 60–67) according to MPCC, with health-related issues affecting the results. The highest level of patient-centredness was achieved in psychological health issues with 79% (65–94), the lowest in respiratory ones with 55% (49–61). Longer consultations resulted in higher MPCC scores.

**Conclusions:**

The level of patient-centredness varied in the addressed health issues as well as in the duration of the consultation. Despite the variation, video interpreting in consultations supports a solid patient-centredness.

**Practice implications:**

We recommend the use of remote video interpreting services for outpatient healthcare to support patient-centred communication and to fill the gap of underrepresentation of qualified interpreters on site, regarding a high diversity of spoken languages.

## Background

Due to several humanitarian crises in 2015 and 2016, the European Union faced a massive increase of asylum seekers, with Germany being a primary destination. More than 1.5 million refugees arrived in Germany between 2014 and 2017 [1]. The majority of refugees came from two countries, namely Syria (38.7%) and Afghanistan (17.9%).

In 2015, more than 40.000 refugees were registered in Hamburg, which was more than three times as many as the year before [2]. In Hamburg, as in other large cities, this resulted in huge challenges such as the provision of accommodation and the supply of basic needs, as well as primary healthcare. Consequently, the federal state of Hamburg established refugee camps, mostly by reorganisation of non-residential facilities into so called first reception centres (FRC). Those accommodations were protected by security personnel and run by social workers. Moreover, primary care walk-in clinics (PCWC) with basic medical equipment were established in those FRCs in order to provide medical care for its residents [3–5]. The PCWC were located in a container on-site with a doctor and a nurse. The patients could present themselves without an appointment during regular consultation hours, depending on the size and occupancy of the FRC. Since 2015 video interpreters have been utilised in medical consultations in Hamburg’s primary care walk-in clinics of first reception centres [6–8].

The healthcare system faced difficulties in providing adequate care due to cultural and linguistic barriers. Factors such as differing cultural understandings of illness, health, and healthcare concepts challenged the provision of healthcare [9, 10]. Language barriers can lead to medical errors, increased utilisation of the healthcare system, lower levels of patient satisfaction, a low level of shared decision-making, and subsequently higher health care costs [11–15]. Reducing linguistic barriers has a positive effect on access to and the quality of medical care [16, 17].

Studies demonstrate that the presence of professional medical interpreters during medical consultations can help to overcome these barriers [18–20]. Video medical interpreting has shown to be both useful [21–25] and equal in quality, regarding patient provider satisfaction and acceptance compared to the more usual in-person interpreters [19, 20, 26–28]. The availability of interpreters is exceedingly difficult due to the high demand of interpreting services, the number of required languages, the local attainability, and the associated costs. Site-independent video interpretation could be a solution to provide adequate communication [29]. In order to provide adequate healthcare, medical doctors in the PCWC were given online access to formal interpreters during their consultations [7]. Data regarding the utilisation of video interpreters in these patient-doctor interactions in refugee camps is scarce. Patient-centredness is a common indicator for the quality of the doctor-patient communication: it is based on “an understanding of the patients’ unique constellation of concerns and subjective experience of illness that is as fundamental to healthcare as physician-defined disease categories” [30]. Moreover, it is a complex and multifaceted approach with three core values:

1. Considering patients’ needs, wants, perspectives and individual experiences,
2. Offering patients opportunities to provide input into and participate in their care, and.
3. Enhancing partnership and understanding in the patient-physician relationship [31, 32].

Patient-centredness is fundamental in intercultural consultations: taking the patient’s perspective, sharing information, and involving the patient in the decision-making process is an even more important challenge when the patient is from a different culture.

In addition, the health needs of refugees often differ from those of the general population in terms of acute psychological health problems, physical problems, and disabilities. Also malnutrition and a wide range of non-communicable and infectious diseases are known to be more prevalent in the population of refugees compared to the general population of the host countries [33–36]. The prevalence of mental distress could be shown to be significantly higher in refugees compared to non-refugees [9].

General population studies in Spain and Sweden showed that the presented health issues as well as the duration of the consultation interrelate with the level of the doctors’ patient-centred communication [37, 38]. In the study by Bodegård et al. [38], consultations were considered less patient-centred by both the patients and the doctors when patients had more than one reason for the visit. In addition, consultations were rated as less patient-centred when patients sought care for reasons other than the purely somatic, such as “mental illness, need of certificate (mainly for sick leave), other administrative tasks, a mix of somatic, mental and/or administrative reasons, or unspecific reasons” [38].

To our knowledge, there are no available data on patient-centredness in (inter-cultural) medical consultations in German primary care walk-in clinics. However, only one study shows the high relevance of various individual aspects of patient-centredness and developable implementation in the German healthcare system from the patients’ perspective. The findings of this article show that patients consider every dimension of patient-centredness as very relevant. However, these seem to only be implemented to some extent in consultations [39].

Our paper explores patient-centred communication in medical consultations with refugees, as well as the main reasons for encounter, its procedures, and the duration of the consultations.

## Methods

### Recruitment

Primary care walk-in clinics at first reception centres were approached with the collaboration of the Public Health department in Hamburg. Two large FRCs were included in the analyses; the average number of patients per month in these FRCs was 911 (SD = 71). One PCWC offered six hours of daily consultations, and the other offered four consultation hours on two separate days each week.

Doctors on duty were informed about the study and asked to participate in videotaped consultations with consenting patients. The patients who came for a medical consultation were introduced to the study in the waiting area of the PCWC by one of the researchers in cooperation with a remote video interpreting service (SAVD Videodolmetschen GmbH) [40], which was already a cooperation partner of the PCWC in Hamburg. The video interpreters were informed about the study in advance by the interpreting service and each consented to the videotaping of the interaction. They were called when their services were needed.

The video interpretation was performed using an additional computer with a wide screen (one in the consultation room and one in a second room for the nurse for pre-consultation questions), showing the interpreter online. The patient was usually seated facing the screen so that both the interpreter and the patient could see each other. The screen could be manually turned off during physical examinations to ensure the patients’ privacy. The videotapes were recorded with a GoPro-video camera. The camera was focused on the interpreting screen to capture all participants, whilst the examination table could not be seen.

Inclusion criteria required the patients to be at least 18 years of age and to be a native speaker in Farsi/Dari or Arabic. Patients were not considered for the study if their health condition was a medical emergency or if they had inabilities preventing them from being surveyed (for example, illiteracy).

The informed consent form and all written information concerning the study were provided in the patients’ native languages mentioned above. Prior to the consultation, patients could ask further questions regarding the study with the help of the video interpreting service.

Our study was conducted from June 2017 to March 2018 with six participating doctors (two male, four female), and three video interpreters (one male, two female). We accumulated 92 consultations of 83 patients for analysis. Patients, interpreters, and doctors were not reimbursed for participating in the study.

### Instruments and procedures

The level of patient-centredness in the videotaped consultations between provider and patient was assessed using the validated rating scheme Measure of Patient-Centered Communication (MPCC) [34–42], highlighting the different aspects of patient-centredness in a detailed coding form with sub scores for thorough investigations. The coding form has three main components:

1. Exploration of the disease and any symptoms, including medical information and attempts of the provider to understand the illness experience of the patient (Grand Total 1),
2. Understanding the whole person by exploring contextual facets such as the patient’s culture, social circumstances, and work (Grand Total 2),
3. Attainment of common ground or a mutual definition of the problem by establishing treatment goals (Grand Total 3).

Interactions were described using a dichotomous (yes/no) format indicating the existence of provider behaviours that were signs of a patient-centred approach. Scores were computed for each of those dimensions of patient-centred communication. The patient overall score was obtained by averaging the three dimension scores and represents the percentage of patient-centred communication ranging from 0 (not at all patient-centred) to 100 (very patient-centred). As the highest total score is 100, results are presented in terms of a percentage. The MPCC coding form with detailed information on the categories can be found in the article by Brown et al. [41].

The interpreting service provided professional and licensed interpreters with either a master’s degree in translation studies or a corresponding qualification if such a master’s degree was not available for their language or a judicial certification [8]. Hence, the statements and questions of the interpreter were coded as patients’ statements and questions.

The duration of the videotaped consultations was measured. The consultations were coded by two raters (physician & research associate) using the original MPCC coding manual by the founders of the measure and thereby following the published coding guidelines [41]. Each videotape was analysed at least twice to fill in gaps in coding. The first 10% of the videotaped consultations were discussed to reassure the same understanding of the MPCC coding form. After that, the videotaped consultations were coded independently. Our average intra-class correlation (ICC) [42, 43] between coders for the overall MPCC was 0.73. The ICCs in other MPCC versions in the literature also show high interrater reliabilities between 0.69 and 0.83 [44].

In order to explore the reasons for encounters and the level of patient-centredness, the main reasons for encounters were assessed by each rater and coded by the International Classification of Primary Care-2nd Edition (ICPC-2) [45]. The mean values of the MPCC scores were calculated for further comparisons.

In addition to the MPCC form, data on patient characteristics (age, gender, nationality) was gathered for the detection of possible correlations during the data analysis.

ICPC-2 procedures − 30, -31, -39, -43, -51, -52, -54, -55, and − 56 were summarised and analysed as “physical examination”, the procedures − 50, -66, -67, and − 68 as “prescription/medical referral” (Table 1).

**Table 1:** Occurring procedures in relation to ICPC-2 categories

**Physical examination**	
-30	Medical Exam/Evaluation-Complete
-31	Medical Examination/Health Evaluation/Pre-op check
-39	Physical Function Test
-43	Other Diagnostic Procedures
-51	Incise/Drain/Flush/Aspirate
-52	Excise/Remove/Biopsy/Destruction/Debride
-54	Repair/Fixate-Suture/Cast/Prosthetic
-55	Local Injection/Infiltration
-56	Dress/Press/Compress/Tamponade
**Prescription/medical referral**	
-50	Medication-Script/Reqst/Renew/Inject
-66	Refer to Other Provider
-67	Referral to Physician/Specialist/Clinic/Hospital
-68	Other Referrals Not Elsewhere Classified

### Statistical methods

For continuous data, mean and standard deviation or median, minimum and maximum were presented. Categorical data was described by absolute and relative frequencies. Patient-centredness and sub scores were described by the mean percentage and 95% confidence interval of the mean. The most frequent ICPC-2 codes, with more than five consultations each, were identified according to the main ICPC-2 chapters. All the other codes were combined in one group named “other”.

The mean MPCC scores in these ICPC-2 groups were calculated and compared using variance analysis (ANOVA), controlled for patient age and gender and for the duration of the consultation as continuously measured (minutes).

MPCC scores were compared for having received versus not having received a physical examination or a medical referral or prescription, respectively by ANOVA, adjusted for age and gender of the patient and for the duration of consultation. Pearson correlations were calculated for the duration of consultation and for the MPCC scores. The correlation coefficient and the p value were reported. Pearson correlations with the MPCC scores were also calculated for the sub groups, according to whether or not they had a physical examination. All p values were calculated two-sidedly. An exploratory data analysis was performed and p values < 0.05 were interpreted as significant outcomes. Statistical calculations were performed with SPSS for windows, version 25 and 29.

## Results

### Sample characteristics

We explored 92 consultations of 83 patients with a mean age of 31 years (Table 2). The majority of the patients were Afghan (N = 45). Nine patients were recorded a second time between two days and ten weeks after their first consultation. Four of the second consultations were with the same doctor.

Three patients presented a health need referring to the same ICPC chapter (digestive, cardiovascular, musculoskeletal) as in the first consultation. All patients may have frequented the PCWC previously.

**Table 2:** Patient characteristics (N = 83)

Age (years)	19 to 65, mean 31, SD 10
Gender	61 male, 22 female
Nationality	45 Afghan 14 Iran 11 Iraqi 7 Syrian 6 of other nationality
Consultations	92, incl. 9 s consultations
Duration of consultations (minutes)	2 to 35, mean 11, SD 7

### Factors associated with patient-centredness (MPCC scores)

The magnitude of patient-centredness according to components and reasons for visit (MPCC) is shown in Table 3. Patient-centredness was most pronounced in “Exploring both the disease and the illness experience” and lowest in “Understanding of the whole person”. The overall score was 64% (95% CI 60–67).

The main health reasons for visit according to ICPC-2 coding were respiratory, musculoskeletal, skin, digestive, and psychological related symptoms (Table 3). The extent of patient-centredness markedly differed regarding the main reason for visit and the “overall score.” The highest patient-centredness was achieved in psychological, the lowest in respiratory symptoms.

**Table 3:** Means and CI95 of MPCC scores by ICPC-2 group, physical examination, and prescription or medical referral

	Exploring both the disease and the illness experience (Grand Total 1)	Understanding of the whole person (Grand Total 2)	Finding common ground (Grand Total 3)	Overall score
*Total (n = 92)*	*71 (68–74)*	*57 (50–63)*	*65 (61–69)*	*64 (60–67)*
**ICPC-2 group**				
Respiratory (R) *(n = 22)* Musculoskeletal (L) *(n = 19)* Skin (S) *(n = 11)* Digestive (D) *(n = 9)* Psychological (P) *(n = 6)* Other *(n = 25)*	68 (62–75) 69 (60–78) 74 (62–86) 74 (65–83) 81 (73–89) 69 (63–76)	42 (28–56) 65 (50–79) 53 (31–76) 55 (38–71) 81 (61–101) 61 (48–73)	55 (48–62) 73 (65–81) 65 (50–80) 65 (54–77) 76 (53–99) 65 (59–72)	55 (49–61) 67 (57–77) 63 (51–75) 65 (58–72) 79 (65–94) 65 (59–72)
F P value	0.821 0.538	1.852 0.112	2.168 0.065	2.370 0.046
**Procedure**				
Physical examination* *No (n = 33)* *Yes (n = 59)*	73 (68–78) 70 (65–74)	65 (55–75) 52 (44–61)	70 (63–76) 62 (58–67)	69 (64–74) 61 (56–65)
F P value	0.676 0.413	3,975 0.049	3.102 0.082	5,832 0.018
Prescription or medical referral** *No (n = 7)* *Yes (n = 85)*	74 (66–82) 71 (67–74)	53 (34–72) 57 (50–64)	62 (45 -79) 65 (61–69)	63 (54–72) 64 (60–68)
F P value	0.601 0.440	0.149 0.701	0.003 0.953	0.439 0.509

MPCC = Measure of Patient-Centered Communication; ICPC-2: International Classification of Primary Care 2nd Edition; CI95 = 95% confidence interval of the mean; numbers are percentages unless otherwise stated; ANOVA = analysis of variance; F-Tests were calculated by ANOVA; correlation coefficients were calculated by Pearson correlations; *including ICPC-2 procedures − 30;-31;-39;-43;-51;-52;-54;-55;-56; ** including ICPC-2 procedures − 50;-66;-67;-68; controlled for patient’s age and gender, and duration of consultation, respectively; duration of consultation was measured once in total (mean 11 min; SD 7); N total = 92 videotaped consultations

A physical examination was performed in 59 (64%) of the consultations. In 85 consultations (92%), the patient received a prescription or a medical referral. Patients received both, an examination as well as a prescription or medical referral, in 57 (62%) of the consultations. Physical examination procedures showed an association with the “overall score” and with “understanding of the whole patient”(Grand Total 2), as shown in Tables 3, with a higher patient-centredness when there was no physical examination. A prescription or medical referral showed no significant differences in our study.

The ANOVAs regarding ICPC-2 and the procedures (physical examination and prescription), showed a gender association in “finding common ground.”

The consultation duration showed an association in the ANOVAs regarding the MPCC scores “understanding of the whole patient,” “finding common ground,” and the “overall score”.

In terms of the duration of the consultation, longer consultations showed higher MPCC scores but longer consulations did not guarantee a high level of patient-centredness. Regarding the duration of the consultation, physical examinations significantly affected the MPCC scores in “understanding of the whole person,” “finding common ground,” and in the “overall score” (Table 4).

**Table 4:** Duration of consultations

	Exploring both the disease and the illness experience (Grand Total 1) *	Understanding of the whole person (Grand Total 2) *	Finding common ground (Grand Total 3) *	Overall score*
**Total**				
Correlation coefficient	0.097	0.393	0.270	0.392
P value	0.356	< 0.001	0.009	< 0.001
… with physical examination** **(n = 59)**				
Correlation coefficient	0.184	0.454	0.309	0.481
P value	0.164	< 0.001	0.017	< 0.001
**… without physical examination (n = 33)**				
Correlation coefficient	-0.099	0.286	0.206	0.232
P value	0.583	0.106	0.251	0.195

*MPCC = Measure of Patient-Centered Communication; Correlation coefficients were calculated by Pearson correlations; **including ICPC-2 procedures − 30;-31;-39;-43;-51;-52;-54;-55;-56.Duration of consultation was measured once in total minutes (mean 11 min; SD 7); N total = 92 videotaped consultations.

## Discussion

In this study, we aimed to investigate the extent of patient-centredness in the consultation process while using video interpreters in outpatient primary care walk in-clinics in two first reception centres in Hamburg. The three MPCC component (Grand Total) ratings of 71%, 57%, 65% and an overall score of 64% demonstrate the level of patient-centredness in the videotaped medical consultations in the PCWCs. Other studies using the MPCC scheme in regular family practice settings have found similar levels of patient-centredness with non-refugee patients [46–48] (Table 5).

**Table 5:** Mean MPCC scores showing “mean scores in percent”

Authors	Clayton et al. 2011 [46]	Mercer et al. 2016 [49]	Bertakis et al.2009 [50]	Our study
Total MPCC (Mean/SD/Range) Overall Score	59 (range 12–85)	42* (SD 16)	50 (SD 8, range 25–74)	64 (SD 17, range 24–100)
**Exploring both the disease and the illness experience** Grand Total 1	42 (range 12–100)	25*(SD 13)	45 (SD 9, range 20–70)	71 (SD 15, range 30–100)
**Understanding of the whole person** Grand Total 2	50 (range 0-100)	29*(38)	50 (SD 19, range 0-100)	57 (SD 31, range 0-100)
**Finding common ground** Grand Total 3	87 (range 0-100)	72 *(18)	54 (SD 12, range 33–100)	65 (SD 18, range 25–100)

MPCC = Measure of Patient-Centered Communication; *Mercer et al. 2016 MPCC scores were categorised as high level deprivation (h) areas. The scores were converted in line with the MPCC scoring system to achieve percentage scores. The score for the standard deviation was equally presumed.

In our study the highest sub score was achieved in “exploring both the disease and the illness experience.” In fact, this value was higher than in most previous studies in family practice settings as presented in Table 5. In contrast, the doctor-patient relationship might not be that well established in first reception centres due to the structure of the setting with rotating doctors and the short stay of the refugees in those centres. Therefore, the patient might feel the need to explain his or her symptoms in more detail. The doctor on his behalf might perform a more thorough exploration and examination. Another important aspect is the intercultural context of these consultations. In those contexts, the perception and associations patients may have about symptoms can differ substantially from the ones that doctors have and might therefore take more time to elaborate. In comparison, only about a quarter of the population in Germany has a migration background, which make family practices a different setting altogether [49]. In the case of those with a migration background, more effort may be required on the part of doctors to understand the perspective of the patient and to integrate the patient more fully in the decision-making process. The latter may also be dependent on the extent of patient-centredness that the patient was culturally accustomed to in the country of origin. Moreover, the spectrum of diseases that doctors are used to in the German population, such as acute psychological health problems or a wide range of non-communicable diseases, may be different than those prevalent in the refugee population. This might be another reason why treating members of the refugee population may take more time.

Interpreters were not present in the studies presented in Table 5. However, the results show that the level of patient-centredness in our setting is similar to the levels in a family practice setting outside of PCWC. Our study is the first one to investigate levels of patient-centredness using the MPCC scheme in this setting. Hence, we assume that this finding might be a valuable source of knowledge for future studies.

Previous studies showed the same level of satisfaction with video interpreting compared to in-person interpreting [29]. Showing a considerable level of patient-centeredness in our study, we conclude that video interpreters support the patient-doctor interaction in medical consultations.

Furthermore, we want to point out that medical staff choosing to work in a refugee setting might be more empathetic or have a more patient-centred approach. This might have an influence on the MPCC scores in our study. It might also reflect an existing positive attitude of the staff towards patient encounters.

The most frequent health reasons for encounters were respiratory, musculoskeletal, skin, digestive, and psychological related symptoms. These symptoms align with the current literature available of patients in first reception centres [4, 5]. Therefore, our paper is consistent with the spectrum of presentations for encounters within the refugee population in Germany.

Our study showed that the level of participation differs between the main reasons for visit.

Regarding the uncertainty of Bodegârd et al. concerning the patient satisfaction in consultations with mental health issues, Fiscella et al. 2004 reported a high level of patient trust and patient-centredness in context with depression [50]. Concordant with Fiscella et al., our study showed higher values in patient-centred communication concerning psychological related symptoms. This was to be expected given the importance of understanding the circumstances of the whole person, patients’ perceptions, and resources to treat a mental illness in comparison to other reasons for the visit. However, there were only six consultations in which the main reason for the encounter was coded as psychological. Possibly, psychologically related reasons for the visit were underrepresented in our study. The fact that our consultations were videotaped might have been a barrier for those patients to participate in the study. On the other hand, psychological problems can also be present in other somatic symptoms (psychosomatic). In our study the lowest scores for patient-centred communication were found in consultations involving respiratory symptoms. The patients’ preference for participation in medical decisions depends upon the kind of health condition, with psychiatric patients often choosing a more active role than patients with chronic somatic disorders, who more often tend to take a passive role [51]. This is congruent with our findings of higher patient-centredness in psychological than in somatic related consultations.

Furthermore, longer consultations resulted in higher MPCC scores. These findings are in accordance with the literature demonstrating that longer consultations tend to be more patient-centred [37]. Longer consultations typically gave room for more patient-centred behaviour, such as allowing the patient to express themselves, even more so when there is little time constraint. Nonetheless, there is a good amount of patient-centred behaviour, also in shorter consultations. The strongest association regarding the duration among the grand totals were shown in “understanding of the whole person”(Grand total 2).

Patient-centredness in consultations with a physical examination showed slightly lower levels compared to those without a physical examination. A physical examination more often resulted from respiratorial and musculoskeletal impairments, which showed lower patient-centredness in our study. One possible reason could be that some patients and especially those with physical complaints may prefer a more directed style in the consultation[52]. Although the direct style can be experienced as patient-centred if this style is preferred by the patient, it often shows lower MPCC values due to the occurrence of comparatively fewer reassurances during the consultation. In contrast, the absence of a physical examination was often related to psychological impairments. In these cases, information is gained with questionnaires and verbal exploration; thus a higher level of patient involvement is mandatory, which in turn results in higher MPCC scores.

On the other hand, the presence or absence of a prescription or medical referral showed no significant effect in our study. One reason could be that only seven patients left without a referral or a prescription. Depending on the patient’s expectation, some interactions do not require a prescription or referral, but rather the sharing of fears or concerns about a condition and the acknowledgement or overcoming of those concerns. In these encounters empathic exploration and discussion of symptoms or fears is a patient-centred approach that will lead to MPCC scores comparable to encounters with a referral or a prescription.

### Limitations

The fact that this study is restricted in the number of participants, doctors, and interpreters as well as by the inclusion of only two PCWCs from first reception centres limits the generalisability of the results. Our study did not use a control group to compare results because video interpreters were already established in all PCWCs in Hamburg during medical consultations prior to our study. Therefore, it would have been considered unethical to withhold consultations with video interpreters from those patients participating in the study and being in the control group.

In addition, we limited the number of languages interpreted to Farsi/Dari and Arabic and omitted those patients who spoke neither language. This was due to the fact that Arabic and Farsi/Dari were the two most commonly spoken languages among patients in the first reception centres during the study period. The established video interpreting service provided interpreters in these languages, as well as translation of the study documents, which increased the feasibility of the study.

We cannot say with certainty whether all reasons for consultation and patient needs were correctly translated by the interpreters in the patients’ interests. It should be noted that information could also have been lost here [53], although the interpreters were certified experts.

Furthermore, the gender ratio was not balanced in our study. Therefore, we controlled for a possible gender effect in the ANOVAs. However, this ratio is similar to the study population of refugees in other PCWCs, for instance in the analysis of Oltrogge et al. with 63% males and 37% females [7]. Finally, we focused on one instrument measuring patient-centredness in doctor-patient communication. Hence the generalisability and transferability of our assessments is limited.

## Conclusions

This study provides first data on the doctor-patient communication in first reception centres and therefore provides new, valuable knowledge in this sector. There is a good patient-centred communication using video interpreting services.

Our study suggests a high acceptance and a good patient involvement in the decision-making process with video interpreting services in all aspects of the consultation. This study provides valuable knowledge on patient-centredness in inter-cultural medical consultations regarding the presented health issues and the actual consultation duration, as there is little research available in this field. Our results suggest that the main reason for a consultation influences the extent of patient-centredness. In our study population, the highest levels were found in consultations with patients presenting with psychological health issues, though the number of those patients was small. Refugees are often traumatised before and during their journey to host countries. Our results showed that these patients can experience a good patient-centred healthcare through PCWCs with the help of a video interpreter, which provides a good basis for further treatment. The fact that longer consultations resulted in higher levels of patient-centredness also suggests that longer consultations might give more opportunities to patient-centred behaviours in general. Further studies are needed on a broader population of refugees in this setting to support our findings.

We recommend the expansion of the use of video interpreting services to the general healthcare system so that patients and doctors can overcome possible language barriers. It could reduce the gap caused by underrepresentation of interpreting services on site, especially in view of the variety of patient spoken languages (refugees, migrants, travellers). This could improve medical care and patient satisfaction as well as reduce the long-term costs for the health care system in outpatient care.

Consultations should possibly be given more time in those settings to gain more patient-centredness for a better outcome for the patient and all those involved.

## Data Availability

The datasets analysed during the current study are available from the corresponding author on reasonable request.

## Declarations

### Co-operations

The co-operating partners, the health department of Hamburg-Altona and “SAVD Videodolmetschen GmbH”, assisted without reimbursement in our study by providing access to the first reception centres, partaking doctors, video interpreters and translation of study material.
